# Items

**Published:** 1860-07

**Authors:** 


### ITEMS.

According to the census of 1859, there were 2,072 physicians in the State of Virginia.

The Legislature of Alabama have made an appropriation of Fifty Thousand Dollars to the Mobile Medical College.

Dr. Brown-Sequard is to deliver a course of lectures on paralysis and epilepsy, at the National Hospital for Epileptics, in London.

Walter V. Wheaton, the Senior Surgeon of the U. S. Army, recently died in Philadelphia, aged 73. He was appointed Hospital Surgeon 1813.

The National Convention for Revising the Pharmacopœia of the United States, held its fifth decennial meeting in the city of Washington, on the 2d of May, 1860.

The Professional friends of Dr. Geo. B. Wood, gave him a complimentary dinner at the Academy of Music on the 16th of May. Dr. W. designs soon to go abroad, to remain for some time.

The Chair of Theory and Practice of Medicine, in the the University of Pennsylvania, made vacant by the resignation of Prof. Geo. B. Wood, has been filled by the election of Dr. William Pepper.

Some Southern periodicals claim that the tea plant flourishes well, in the climate of Louisiana, enduring equally well the hotest days of summer and the frosts of autumn.

The Legislature of Massachusetts has been convened to take such action as is thought most effectual to prevent the extension of the disease among cattle that is prevailing in that State. The opinion seems to be held, that the malady spreads only by contagion.

